# European validation of the Barcelona magnetic resonance predictive model for significant prostate cancer detection in prostate biopsies

**DOI:** 10.1002/bco2.70198

**Published:** 2026-04-10

**Authors:** Juan Morote, Berta Miró, Lucas Regis, François Cousin, Patrick Duflot, Philippe Kolh, Alice Andalò, Nicola Gentili, Filippo Merloni, Andrea Prochowski Iamurri, Fabio Ferroni, Enrique Trilla, Olga Méndez

**Affiliations:** ^1^ Department of Surgery Universitat Autònoma de Barcelona Bellaterra Spain; ^2^ Department of Urology Vall d'Hebron University Hospital Barcelona Spain; ^3^ Research Group of Urology Vall d'Hebron Research Institute Barcelona Spain; ^4^ Statistics Unit Vall d'Hebron Research Institute Barcelona Spain; ^5^ Department of Oncological Imaging Liège University Hospital Liège Belgium; ^6^ Department of Medical Information Systems Liège University Hospital Liège Belgium; ^7^ Data Unit IRCCS Istituto Romagnolo per lo Studio dei Tumori (IRST) “Dino Amadori” Meldola Italy; ^8^ Medical Oncology Unit IRCCS Istituto Romagnolo per lo Studio dei Tumori (IRST) “Dino Amadori” Meldola Italy; ^9^ Radiology Unit IRCCS Istituto Romagnolo per lo Studio dei Tumori (IRST) “Dino Amadori” Meldola Italy

**Keywords:** early detection, federated network, predictive models, significant prostate cancer, validation

Early detection of significant prostate cancer (sPCa) has notably improved with the widespread use of magnetic resonance imaging (MRI) and targeted biopsies. This advancement has contributed to the recommendation for PCa screening in the European Union. Nevertheless, PCa suspicion is still primarily based on a serum prostate‐specific antigen (PSA) level greater than 3.0 ng/mL and/or a suspicious digital rectal examination (DRE). Prostate MRI is then requested to identify lesions suspected of harbouring sPCa, particularly when the Prostate Imaging‐Reporting and Data System (PI‐RADS) score is above 2. In such cases, both targeted biopsies of suspicious lesions and systematic biopsies are recommended, whereas prostate biopsy is typically avoided when the PI‐RADS score is below 3. The European Association of Urology recommends the use of predictive models to reduce unnecessary prostate biopsies and the over‐detection of insignificant prostate cancer (iPCa), especially in uncertain scenarios as PI‐RADS 3 where the mean rate of sPCa detection is 20% and the overdetection of iPCa remains up to 50%.^1^ Accessible web‐ or smartphone‐based risk calculators are essential for integrating predictive models into routine practice for assessing individual sPCa likelihood. Furthermore, validating these predictive models in populations different from those used in their development is crucial to ensure generalizability and clinical reliability.^2^

The Barcelona MRI predictive model (BCN‐MRI PM) was developed at a single academic institution within 1487 men suspected of having PCa, drawn from the sPCa opportunistic early detection program of Catalonia between 2016 and 2019. Two‐ to four‐core MRI‐transrectal ultrasound fusion targeted biopsies of PI‐RADS lesions >2 and/or 12‐core systematic biopsies when PI‐RADS <3 were conducted via the transrectal route. This model, developed using logistic regression, incorporated age (years), family history of PCa (no vs. yes), type of prostate biopsy (initial vs. repeated), serum PSA level (ng/mL), DRE (normal vs. suspicious), MRI‐derived prostate volume (mL) and PI‐RADS v 2.0 (1–5) as independent predictive variables for sPCa. An initial external validation was conducted in 946 men, from two other institutions within the same metropolitan area, using the same criteria for PCa suspicion and diagnostic approach.^2^ Subsequent validations of the BCN‐MRI PM have been conducted in patients with symptomatic benign prostatic hyperplasia undergoing 5‐alpha reductase inhibitors,^3^ men classified with the PI‐RADS v 2.1 and subjected to transperineal prostate biopsies^4^ and men subjected to transperineal mapping biopsies of suspicious lesions and perilesional areas.^5^

Federated learning is emerging as a critical strategy for enabling the online validation of predictive models by utilizing continuous feedback from local new cases and appropriate machine learning algorithms. We hypothesize that validation of the BCN‐MRI PM can be further developed within the framework of the **F**ederated **L**earning and m**U**lti‐party computation **T**echniques for Prostat**E** Cancer (FLUTE) project. This initiative involves three clinical partners across Spain, Belgium and Italy. A key feature of this project is that data are managed locally through the FLUTE platform.^6^ Although this study does not yet implement a full federated learning pipeline, it represents a foundational step: The model was independently validated at three clinical partner sites using local data, consistent with the FLUTE logic of decentralized analysis.

The validation was successfully conducted at Vall d'Hebron Research Institute in Barcelona, Spain (VHIR); Centre Hospitalier Universitaire in Liège, Belgium (CHUL); and IRCCS Istituto Romagnolo per lo Studio dei Tumori (IRST) “Dino Amadori,” Meldola, Italy (IRST).^6^ Approval from each ethical committee was obtained: VHIR (2024/150), CHUL (2024/133) and IRST (3587/2024). Data from 3557 participants was collected at VHIR, from 672 at CHUL and 105 at IRST. All participants were retrospectively selected from men suspected of having PCa who underwent multiparametric MRI and targeted biopsies of PI‐RADS v 2.1 lesions ≥3 and/or systematic biopsies when PI‐RADS <3, within 2021 and 2024. Only cases with complete data on the seven variables required by the BCN‐MRI PM were included. Men with previously detected PCa, ASAP or HG‐PIN were excluded as in the development cohort, and MRIs were reported at each site.^2^ PCa suspicion criteria were consistent across all centres. The sPCa detection rates were 43.8% at VHIR, 53.3% at CHUL and 34.3% at IRST (Table S1). There were notable differences in patient characteristics (Table S2) and diagnostic or biopsy approaches (Table S3), although all centres adhered to the European PCa guidelines. sPCa was defined as an International Society of Urological Pathology grade group ≥ 2.^7^

Data processing was performed locally at each participant centre. Individual sPCa likelihoods were assessed using Python (Python software Foundation; http://python.org), based on the original BCN‐MRI PM provided by VHIR. The rate of sPCa detection according to the PI‐RADS score at each centre is summarized in Table S4. Calibration was assessed using calibration plots comparing predicted and observed probabilities, showing good overall agreement across centres. Discrimination of sPCa, evaluated using the receiver operating characteristic (ROC) curves, is presented in Figure 1A–C. The area under curve (AUC) was 0.83 (95% CI: 0.81–0.85) at VHIR, 0.77 (95% CI: 0.73–0.81) at CHUL and 0.71 (95% CI: 0.61–0.80) at IRST (Table S5). Decision curve analysis (DCA) was used to assess the clinical usefulness of the BCN‐MRI PM by quantifying its net benefit across a range of clinically relevant threshold probabilities. Unlike traditional performance metrics, DCA explicitly accounts for the trade‐off between correctly identifying men suspected of having sPCa who would benefit from a prostate biopsy and the harm of unnecessary biopsies. DCA results are interpreted by comparing the net benefit of the model with default strategies such as ‘treat all’ and ‘treat none’; a model is considered clinically useful within the range of threshold probabilities where it provides a higher net benefit than these alternatives, indicating that its use would lead to better decision‐making in clinical practice.^8^ DCA analysis showed greater net clinical benefit of the BCN MRI‐PM model over biopsying all men only above centre‐specific risk thresholds (≈10% at VHIR and CHUL, ≈20% at IRST), highlighting threshold‐dependent clinical utility (Figure 1D–F). Clinical efficacy was further assessed using clinical utility curves (CUCs), which describe the trade‐off between avoided biopsies and missed sPCa across threshold probabilities (Figure 1G–I). At a sensitivity of approximately 95%, use of the BCN MRI‐PM model avoided 21% of biopsies at VHIR and 19% at CHUL. At IRST, the closest achievable operating point yielded 91.7% sensitivity with 16.2% of biopsies avoided (Table S6).

**FIGURE 1:**
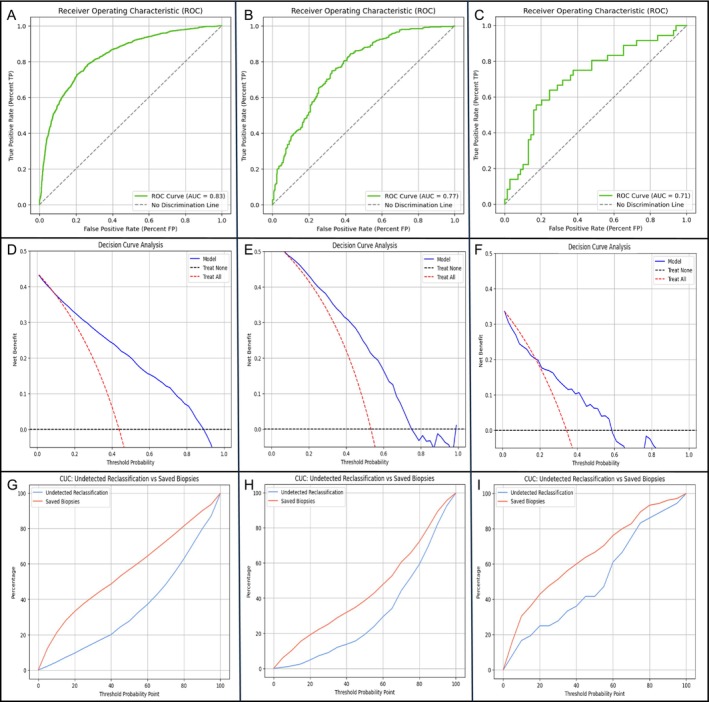
Discrimination ability of the BCN‐MRI PM for sPCa discrimination at VHIR (A), CHUL (B) and IRST (C). Net benefit of the BCN‐MRI PM over biopsying all men at VHIR (D), CHUL (E) and IRST (F). Clinical utility curves showing the proportion of avoided prostate biopsies and the rate of undetected sPCa across the continuous threshold probability from 0% to 100% at VHIR (G), CHUL (H) and IRST (I).

The BCN‐MRI PM discrimination ability for detecting sPCa showed comparable performance, with AUCs ranging from 0.71 to 0.83 across the centres. Only the AUCs for VHIR and CHUL showed a minor but statistically significant difference (adjusted *p* = 0.03). Clinical utility varied between centres: The model avoided 16.2%–21% of biopsies while missing 4.5%–8.7% of sPCa cases. Variability in model performance across centres may be attributed to differences in population characteristics, particularly the distribution of PI‐RADS categories and rates of sPCa detection in each centre. Differences in diagnostic protocols may also explain the varying clinical utility, especially as sPCa detection rates ranged from 34.3% to 53.3%. The large variation in sPCa prevalence across the three centres precludes a meaningful comparison of the three DCAs.

This study is limited by cohort heterogeneity, the lack of centralized radiology reports and differences in sPCa detection rates. Nevertheless, such heterogeneity represents a robust test for the BCN‐MRI PM. Potential selection bias exists due to the exclusion criteria. Additionally, since biopsy‐based detection does not reflect the true pathology of the entire prostate gland, the true clinical performance may vary. Advanced statistical analyses across centres were not feasible due to the absence of centralized data. Beyond validating an MRI‐based predictive model, it is recommended to assess whether it achieves 100% sensitivity in PI‐RADS 4–5, as its use would not be justified if any sPCa were missed with these categories.^2^

This study represents an essential first step toward achieving continuous online validation of the BCN‐MRI PM at each FLUTE clinical partner site. The project also plans to incorporate radiomic analysis of MRI exams.^9^ Future iterations of the BCN‐MRI PM, incorporating artificial intelligence in a federated setting, are expected to further improve sPCa discrimination and enable ongoing validation at each participating centre.

## AUTHOR CONTRIBUTIONS

Juan Morote, Berta Miró and Olga Méndez conceptualized the idea. Juan Morote Berta Miró, Lucas Regis, Enrique Trilla, Olga Méndez, François Cousin, Patrick Duflot, Philippe Kolh, Alice Andalò, Nicola Gentili, Fillipo Merloni, Andrea Prochowskiiamurri and Fabio Ferroni developed the concept. Juan Morote wrote the first draft of the manuscript. All authors were involved in editing, critical review and final approval of the manuscript.

## CONFLICT OF INTEREST STATEMENT

The authors have no conflict of interest to declare.

## Supporting information


**Table S1** Characteristics of the populations participating in the validation of de BCN MRI predictive model.
**Table S2** Statistical comparison of baseline characteristics across the VHIR, CHUL, and IRST cohorts. This table summarizes the pairwise differences and associated p‐values for the variables described in Supplementary Table 1. Continuous variables were compared using *t*‐tests; categorical variables were compared using proportion *z*‐tests. Distributional differences in PI‐RADS scores were assessed using chi‐squared tests.
**Table S3** Characteristics of PCa suspicion, diagnostic approach and definition of sPCa in prostate biopsy applied at the three participant centers.
**Table S4** Rate of sPCa detection according to the PI‐RADS score in the three participant centers.
**Table S5** Pairwise comparison of AUCs for the VHIR, CHUL, and IRST cohorts. The table shows the raw and Bonferroni‐adjusted p‐values for the differences between centers.
**Table S6** Undetected sPCa and avoided prostate biopsies corresponding to each threshold of the BCN‐MRI predictive model in the participant sites.
