# Supplementary material for: European validation of the Barcelona magnetic resonance predictive model for significant prostate cancer detection in prostate biopsies
Source: BJUI Compass. 2026 Apr 10;7(4):e70198. [Article in Spanish] doi: 10.1002/bco2.70198 (PMC13066910; doi:10.1002/bco2.70198)
Supplement: Supplementary file 1 — Table S1 Characteristics of the populations participating in the validation of de BCN MRI predictive model. Table S2 Statistical comparison of baseline characteristics across the VHIR, CHUL, and IRST cohorts. This table summarizes the pairwise differences and associated p‐values for the variables described in Supplementary Table 1. Continuous variables were compared using t‐tests; categorical variables were compared using proportion z‐tests. Distributional differences in PI‐RADS scores were assessed using chi‐squared tests. Table S3 Characteristics of PCa suspicion, diagnostic approach and definition of sPCa in prostate biopsy applied at the three participant centers. Table S4 Rate of sPCa detection according to the PI‐RADS score in the three participant centers. Table S5 Pairwise comparison of AUCs for the VHIR, CHUL, and IRST cohorts. The table shows the raw and Bonferroni‐adjusted p‐values for the differences between centers. Table S6 Undetected sPCa and avoided prostate biopsies corresponding to each threshold of the BCN‐MRI predictive model in the participant sites. [file BCO2-7-e70198-s001.docx]

**Supplementary Table 1** Characteristics of the populations participating in the validation of de BCN MRI predictive model.

| **Characteristic** | **VHIR** | **CHUL** | **IRST** |
| --- | --- | --- | --- |
| Number of men | 3,557 | 672 | 105 |
| Mean age, years (SD) | 67.9 (8.0) | 66.5 (6.7) | 66.5 (8.5) |
| Mean serum PSA, ng/mL (SD) | 14.0 (36.2) | 12.7 (29.8) | 16.7 (47.9) |
| Abnormal DRE, n (%) | 1102 (29.61%) | 217 (31.65%) | 13 (14.7%) |
| PCa family history, n (%) | 225 (6.4) | 90 (13.5%) | 10 (8.6%) |
| Previous negative prostate biopsy, n (%) | 1,125 (30.2%) | 155 (23.7%) | 47 (42%) |
| Mean prostate volume, mL (SD) | 60.4 (31.2) | 55.8 (31.8) | 66.9 (35.8) |
| PI-RADS, n (%) |  |  |  |
| 1 | 341 (7.10%) | 16 (2.55%) | 0 (0%) |
| 2 | 102 (2.07%) | 56 (8.8%) | 21 (16.5%) |
| 3 | 825 (17.64%) | 100 (15.3%) | 20 (17.8%) |
| 4 | 1573 (44.52%) | 245 (36.45%) | 45 (43.2%) |
| 5 | 716 (15.53%) | 255 (37%) | 19 (22.4%) |
| sPCa detection, n (%) | 1,559 (43.8%) | 358 (53.3%) | 36 (34.3%) |
| *VHIR: Vall Hebron Research Institute; CHUL: Centre Hospitalier Universitaire de Liège; IRST: Instituto Romagnolo per lo Studio dei Tumori; sPCa: significant prostate cancer; SD: standard deviation; PSA: Prostate specific antigen; DRE: digital rectal examination; PCa: prostate cancer; sPCa: significant PCa; PI-RADS: Prostate imaging-reporting and data system.* | | | |

**Supplementary Table 2** Statistical comparison of baseline characteristics across the VHIR, CHUL, and IRST cohorts. This table summarizes the pairwise differences and associated p-values for the variables described in Supplementary Table 1. Continuous variables were compared using t-tests; categorical variables were compared using proportion z-tests. Distributional differences in PI-RADS scores were assessed using chi-squared tests.

| **Characteristic** | **Comparison** | ***p**** |
| --- | --- | --- |
| Mean age, years | VHIR vs. CHUL | <0.0001 |
|  | VHIR vs. IRST | =0.0962 |
| Mean serum PSA, ng/mL | CHUL vs. IRST | =0.9542 |
|  | VHIR vs. CHUL | =0.3145 |
|  | VHIR vs. IRST | =0.5698 |
| Abnormal DRE | CHUL vs. IRST | =0.4082 |
|  | VHIR vs. CHUL | =0.5012 |
|  | VHIR vs. IRST | <0.0001 |
| PCa family history | CHUL vs. IRST | <0.0001 |
|  | VHIR vs. CHUL | <0.0001 |
|  | VHIR vs. IRST | =0.1875 |
| Previous negative biopsy | CHUL vs. IRST | =0.2709 |
|  | VHIR vs. CHUL | <0.0001 |
|  | VHIR vs. IRST | =0.0045 |
| Mean prostate volume, mL | CHUL vs. IRST | <0.0001 |
|  | VHIR vs. CHUL | =0.0007 |
|  | VHIR vs. IRST | =0.0668 |
|  | CHUL vs. IRST | =0.0033 |
| PI-RADS (distribution 1-5) | VHIR vs. CHUL | <0.0001 |
| PI-RADS (distribution 2-5) | VHIR vs. IRST | =0.0180 |
| PI-RADS (distribution 2-5) | CHUL vs. IRST | =0.0080 |
| sPCa detection | VHIR vs. CHUL | <0.0001 |
|  | VHIR vs. IRST | =0.0519 |
|  | CHUL vs. IRST | =0.0003 |
| **In comparisons involving IRST, PI-RADS category 1 was excluded due to missing data. All tests were based on available aggregated data; no individual-level data were used.*  *VHIR: Vall Hebron Research Institute; CHUL: Centre Hospitalier Universitaire de Liège; IRST: Instituto Romagnolo per lo Studio dei Tumori; sPCa: significant prostate cancer: PSA: Prostate specific antigen; DRE: digital rectal examination; sPCa: significant prostate cancer; PI-RADS: Prostate imaging-reporting and data system.* | | |

**Supplementary Table 3** Characteristics of PCa suspicion, diagnostic approach and definition of sPCa in prostate biopsy applied at the three participant centers

| **Characteristic** | **VHIR** | **CHUL** | **IRST** |
| --- | --- | --- | --- |
| **PCa suspicion** |  |  |  |
| PSA > 3 ng/mL and/or +DRE | Yes | Yes | Yes |
| **PCa diagnostic procedures** |  |  |  |
| mpMRI strength, Tesla | 3.0 | 3.0 | 3.0 |
| Targeted biopsy of lesions PI-RADS > 3 | Yes | No | Yes |
| Targeted biopsy of only the index lesion | No | Yes | No |
| Number of cores in each targeted biopsy | 2-4 | 2 | 4 |
| Systematic biopsy | Yes | Yes | Yes |
| Number of cores of systematic biopsy | 12 | 12 | 12 |
| Fusion biopsy | Yes | Yes | Yes |
| Cognitive fusion biopsy (%) | 70 | 100 | 0 |
| Software fusion biopsy (%) | 30 | 0 | 100 |
| Transrectal biopsy route (%) | 60 | 100 | 100 |
| Transperineal biopsy route (%) | 40 | 0 | 0 |
| **sPCa definition in prostate biopsy, GG** | 2-5 | 2-5 | 2-5 |
| *VHIR: Vall Hebron Research Institute; CHUL: Centre Hospitalier Universitaire de Liège; IRST: Instituto Romagnolo per lo Studio dei Tumori; PCa: prostate cancer; sPCa: significant PCa; mpMRI: multiparametric magnetic resonance imaging; PI-RADS: Prostate imaging-reporting and data system; GG: grade group.* | | | |

**Supplementary Table 4** Rate of sPCa detection according to the PI-RADS score in the three participant centers

| **PIRADS SCORE** | **VHIR** | | **CHUL** | | **IRST** | |
| --- | --- | --- | --- | --- | --- | --- |
|  | **n** | **%** | **n** | **%** | **n** | **%** |
| 1 | 54/341 | 15.8 | 1/16 | 6.3 | 0/0 | 0 |
| 2 | 16/102 | 15.7 | 7/56 | 12.5 | 2/21 | 9.5 |
| 3 | 142/825 | 17.2 | 32/100 | 32.0 | 5/20 | 25.0 |
| 4 | 759/1,573 | 48.3 | 133/245 | 54.3 | 16/45 | 35.6 |
| 5 | 588/716 | 82.1 | 185/255 | 72.6 | 13/19 | 68.4 |
| *VHIR: Vall Hebron Research Institute; CHUL: Centre Hospitalier Universitaire de Liège; IRST: Instituto Romagnolo per lo Studio dei Tumori; n: number of sPCa/ number of cases; %: rate of sPCa.* | | | | | | |

**Supplementary Table 5** Pairwise comparison of AUCs for the VHIR, CHUL, and IRST cohorts. The table shows the raw and Bonferroni-adjusted p-values for the differences between centers.

| **Institution** | **AUCs** | | ***Raw p*** | ***Adjusted p*** |
| --- | --- | --- | --- | --- |
| VHIR vs. CHUL | 0.83 | 0.77 | =0.0115 | =0.0345 |
| VHIR vs. IRST | 0.83 | 0.71 | =0.0176 | =0.0528 |
| CHUL vs. IRST | 0.77 | 0.71 | =0.2659 | =0.7977 |
| *VHIR: Vall Hebron Research Institute; CHUL: Centre Hospitalier Universitaire de Liège; IRST: Instituto Romagnolo per lo Studio dei Tumori; AUCs: areas under the curve* | | | | |

**Supplementary Table 6** Undetected sPCa and avoided prostate biopsies corresponding to each threshold of the BCN-MRI predictive model in the participant sites.

| **Threshold**  **%** | **VHIR** | | **CHUL** | | **IRST** | |
| --- | --- | --- | --- | --- | --- | --- |
|  | **Undetected sPCa** | **Avoided PB** | **Undetected sPCa** | **Avoided PB** | **Undetected sPCa** | **Avoided PB** |
| 5 | 2,1 | 12,2 | 0,6 | 6,0 | 8,3 | 16,2 |
| 10 | 4,5 | 21,0 | 1,4 | 10,3 | 16,7 | 30,5 |
| 15 | 7,2 | 27,9 | 2,5 | 15,5 | 19,4 | 36,2 |
| 20 | 9,6 | 33,1 | 4,7 | 19,0 | 25,0 | 42,9 |
| 25 | 12,3 | 37,6 | 7,3 | 22,3 | 25,0 | 47,6 |
| 30 | 14,9 | 41,5 | 8,9 | 25,1 | 27,8 | 51,4 |
| 35 | 17,5 | 45,3 | 12,0 | 28,9 | 33,3 | 56,2 |
| 40 | 20,0 | 48,6 | 13,7 | 31,8 | 36,1 | 60,0 |
| 45 | 24,3 | 53,0 | 15,6 | 34,8 | 41,7 | 63,8 |
| 50 | 27,6 | 56,8 | 19,3 | 38,5 | 41,7 | 66,7 |
| 55 | 32,6 | 60,5 | 23,7 | 42,7 | 47,2 | 70,5 |
| 60 | 37,1 | 64,4 | 29,3 | 47,8 | 61,1 | 76,2 |
| 65 | 42,5 | 68,6 | 34,1 | 52,7 | 66,7 | 80,0 |
| 70 | 48,4 | 72,7 | 44,1 | 60,4 | 75,0 | 82,9 |
| 75 | 55,4 | 77,0 | 51,4 | 65,5 | 83,3 | 89,5 |
| 80 | 62,9 | 81,4 | 59,2 | 72,2 | 86,1 | 93,3 |
| 85 | 71,3 | 85,7 | 69,8 | 80,5 | 88,9 | 94,3 |
| 90 | 79,7 | 90,0 | 82,1 | 89,3 | 91,7 | 96,2 |
| 95 | 87,1 | 93,7 | 92,5 | 95,5 | 94,4 | 97,1 |
| 100 | 99,7 | 99,9 | 99,7 | 99,9 | 100,0 | 100,0 |
| *VHIR: Vall Hebron Research Institute; CHUL: Centre Hospitalier Universitaire de Liège; IRST: Instituto Romagnolo per lo Studio dei Tumori; sPCa: significant prostate cancer: PB: prostate biopsies* | | | | | | |
